# Wanted, Lady Mission Doctors

**Published:** 1908-01-25

**Authors:** H. H. Montgomery


### WANTED, LADY MISSION DOCTORS.

Sir,—You have from time to time been good enough to make special missionary needs known through your columns. Will you now give publicity to the urgent need for more women doctors in the foreign field? Pioneer work waits to be begun in at least two districts of India; an overworked doctor in China needs a colleague, and now the grievous news of the death on January of Dr. Marie Hayes at Delhi makes the reinforcement of the staff of that Mission a matter of urgent necessity. In a letter dated weeks ago she said herself: "We desperately need another doctor."

In two years from now there will, as we hope, be doctors qualified who are now going through the medical schools with this end in view. But these needs are immediate. We appeal earnestly for two medical women already qualified, and able to undertake responsible work, who would offer at once for this work, especially for Delhi.

For particulars apply to the C.W.W. Candidates Secretary, S.P.G. House, 19 Delahay Street, S.W.

Yours,

H. H. Montgomery (Bishop).

January 20, 1908.

